# Constitutive Modeling of Annealed OFHC with Wide Strain-Rate and Temperature Effects: Incorporating Dislocation Dynamics and Normalized Microstructural Size Evolution

**DOI:** 10.3390/ma16196517

**Published:** 2023-09-30

**Authors:** Mengwen Xu, Qiangqiang Xiao, Xudong Zu, Yaping Tan, Zhengxiang Huang

**Affiliations:** 1 School of Mechanical Engineering, Nanjing University of Science and Technology, Nanjing 210094, China; 2 School of Information Technology, Jiangsu Open University, Nanjing 210094, China

**Keywords:** constitutive modelling, microstructural sensitive, OFHC copper, high strain rate

## Abstract

The flow stress of face-centered cubic (FCC) metals exhibits a rapid increase near a strain rate of 10^4^ s^−1^ under fixed-strain conditions. However, many existing constitutive models either fail to capture the mechanical characteristics of this plastic deformation or use piecewise strain-rate hardening models to describe this phenomenon. Unfortunately, these piecewise models may suffer from issues such as discontinuity of physical quantities and difficulties in determining segment markers, and struggle to reflect the underlying physical mechanisms that give rise to this mutation phenomenon. In light of this, this paper proposes that the abrupt change in flow stress sensitivity to strain rate in FCC metals can be attributed to microstructural evolution characteristics. To address this, a continuous semiempirical physical constitutive model for FCC metals is established based on the microstructural size evolution proposed by Molinari and Ravichandran and the dislocation motion slip mechanism. This model effectively describes the mutation behavior of strain-rate sensitivity under fixed strain, particularly evident in an annealed OFHC. The predicted results of the model across a wide range of strain rates (10^−4^–10^6^ s^−1^) and temperatures (77–1096 K) demonstrate relative errors generally within ±10% of the experimental values. Furthermore, the model is compared with five other models, including the mechanical threshold stress (MTS), Nemat-Nasser–Li (NNL), Preston–Tonks–Wallace (PTW), Johnson–Cook (JC), and Molinari–Ravichandran (MR) models. A comprehensive illustration of errors reveals that the proposed model outperforms the other five models in describing the plastic deformation behavior of OFHC. The error results offer valuable insights for selecting appropriate models for engineering applications and provide significant contributions to the field.

## 1. Introduction

Face-centered cubic (FCC) metals, such as aluminum and copper, are widely utilized in a variety of civil and military applications [1]. However, during processes involving high strain rates and large strains, such as impact, penetration, and deformation processing, these materials exhibit complex nonlinear dynamic mechanical behavior [2]. This behavior encompasses a broad range of strain rates and temperatures, resulting in significant coupling between strain rate, temperature, and strain hardening [3,4]. Moreover, it is influenced by the historical effects of strain rate and temperature, presenting a formidable challenge in accurately constructing plastic constitutive models.

To address these challenges, various dynamic plastic constitutive models have been proposed in recent decades. These models can be classified into two main categories [1]: phenomenological models and models incorporating microstructural evolution. Phenomenological models are purely mathematical and conform to physical laws while possessing simple forms. They require fewer parameters to be calibrated and can produce accurate results within the calibrated range of plastic deformation. The Johnson–Cook (JC) model [5] is one of the most widely used phenomenological constitutive models that consider strain hardening and rate–temperature effects. It is based on the overstress type of models introduced by Malvern [6], Perzyna [7], and Campbell [8] and has been modified. Other variations of the JC model [9,10,11,12] have also been proposed using experimental data. However, these models may deviate significantly from reality for plastic deformation beyond the calibrated range of materials.

Experimental studies by Follansbee and Kocks [13] have shown that certain FCC metals, such as annealed oxygen-free high conductivity copper (OFHC), exhibit a sharp increase in flow stress when the strain rate exceeds a critical value (~10^4^ s^−1^). Despite the high strain rate, plastic deformation remains dominated by the thermal activation of dislocation motion. This remarkable rate sensitivity is primarily attributed to the microstructural evolution during deformation, which is not accounted for in phenomenological models. Microstructural evolution refers to the changes in the arrangement and characteristics of dislocations at the microscale [14]. These changes can include a rapid increase in dislocation density and a significant decrease in characteristic lengths of dislocations (e.g., dislocation spacing, thermally activated area, cell size), leading to a rapid rise in flow stress [15,16]. Recently, Lea’s research [17,18] highlights the paramount significance of microstructure and composition as the primary determinants leading to substantial variations in the dynamic mechanical response of copper under high strain rates, despite minor variations in purity. As a result, it is imperative to consider the impact of microstructural evolution on a material’s mechanical performance when modeling.

In response to the influence of microstructural evolution on material dynamics, several constitutive models have been proposed [15]. These models postulate that the storage distance of dislocations restricts their range of motion, resulting in a limited displacement that dislocations can undergo in unit time. This limitation increases the possibility of interactions between dislocations and defects, such as solutes and vacancies, leading to the accumulation and pileup of more dislocations and enhancing the strain hardening effect of the material. Examples of such models include the Mecking–Kocks (MK) model [14], Zerilli–Armstrong (ZA) model [19], mechanical threshold stress (MTS) model [13], Nemat-Nasser–Li (NNL) model [20], Gao–Zhang (GZ-1, GZ-2) models [3,21], and Austin–McDowell models [22,23]. Among these models, the MTS model [13] captures the mutation phenomenon of strain hardening sensitivity through a single “continuity” equation with the Voce law. However, this model requires various experimental data and phenomenologically based expressions to determine the thermal component of the material’s behavior, which cannot be explicitly stated, leading to a complex constitutive form that can be challenging to manipulate. On the other hand, the Preston–Tonks–Wallace (PTW) model [24] incorporates an enhanced Voce strain hardening criterion to characterize the rate-sensitive mutation phenomenon in materials. It employs piecewise functions within the intermediate regime (10^4^ s^−1^ to 10^8^ s^−1^) to depict thermally activated dislocation glide mechanisms or phonon-drag mechanisms, which may result in a discontinuous strain-rate sensitivity of stress ($m=\partial ln\sigma /\partial ln\dot{\varepsilon }$) with fixed strain. Similarly, the GZ-2 model [3] also employs piecewise functions to characterize the strain-rate sensitivity mutations, resulting in discontinuous strain-rate sensitivity coefficients. The abovementioned constitutive models provide valuable insights into the effects of microstructural evolution on the strain hardening behavior of FCC metals, contributing to the development of accurate predictive models.

In addition to phenomenological and microstructure-based models, semiempirical physical models have also been developed. These models combine experimental data with physical principles and employ empirical parameters or fitting coefficients to simulate the evolution of microstructure-related internal variables. One notable model in this regard is the Molinari–Ravichandran (MR) model [25], which successfully predicts the plastic flow behavior of annealed copper across a wide range of strain rates spanning seven orders of magnitude. The MR model introduces a single internal variable representing the effective microstructural length related to temperature and strain rate, offering valuable insights for metal microstructure design. Other extensions of the MR model are proposed by Durrenberger et al. [26,27], considering the flow stress as the sum of internal stress (long-range interaction) and thermally activated effective stress, providing simplicity and flexibility in describing thermoviscoplastic behavior under extreme loading conditions. However, their application under high-temperature working conditions still needs improvement.

Motivated by the cell characteristic size evolution mechanism in the MR model, this study develops a simplified semiempirical physical constitutive model with a single “continuity” equation to capture stress upturning. It utilizes a normalized cell size evolution equation and incorporates the thermal activation of dislocation motion to capture the plastic dynamic mechanical behavior of FCC metals across a wide range of strain rates and temperatures. In this paper, a comprehensive explanation of the theoretical modeling methods for the components of thermal and athermal stresses in the flow stress is presented in Section 2. Section 3 provides the constitutive parameters for annealed OFHC and the sources of experimental values used to validate the predictive capability of the models. Additionally, a comparative analysis is conducted between the proposed model and other mature models, including the MTS, NNL, PTW, JC, and MR models, to evaluate their performance in predicting experimental results (Section 4). Finally, the main conclusions are summarized in Section 5.

This study aims to contribute to the advancement of constitutive modeling by capturing the complex behavior of materials during plastic deformation. By incorporating the microstructural evolution and the thermal activation of dislocation motion, the proposed model provides valuable insights into the dynamic mechanical behavior of FCC metals under various strain rates and temperatures.

## 2. Constitutive Modelling

Plastic flow during the thermally activated motion of dislocations is primarily governed by the movement of dislocations, which will encounter obstacles in the form of short-range and long-range barriers during their motion. Long-range barriers are nontemperature dependent and are influenced by factors such as grain boundaries, far-field forest dislocations, and other microstructural elements [1,20]. These barriers can impact dislocation motion through mechanisms such as externally applied stress or chemical potential differences. Conversely, the height of short-range barriers is closely related to temperature. At absolute zero temperature (0 K), the height of short-range barriers reaches its maximum value because there is no thermal energy available for dislocations to overcome them. However, the atomic vibration amplitude in the material increases with temperature rising, thereby reducing the height of the short-range barriers. This increase in thermal energy assists dislocations in overcoming the short-range barriers [1]. Therefore, short-range barriers can be overcome through a thermal activation mechanism. In FCC metals, short-range barriers may include forest dislocations, point defects (such as vacancies and self-interstitials), solute atoms, impurities, and precipitates. The presence of these short-range barriers plays a significant role in plastic deformation and the mechanical properties of materials. Thus, based on the nature of barriers and the theory of MTS, the flow stress of a material can be expressed as [13]

(1) $$\sigma =\sigma_{ath}+\sigma_{th}=\sigma_{ath}+\Phi \left(\dot{\varepsilon },\;T\right)\cdot \;{\hat{\sigma }}_{th}$$

where $\sigma_{ath}$ represents an athermal component of flow stress (rate independent), which is influenced by material structure properties. $\sigma_{th}$ represents a thermal component of flow stress (rate dependent), which characterizes the resistance overcome by mobile dislocations through diffusion or slip, driven by thermal activation effects, leading to material plastic deformation. ${\hat{\sigma }}_{th}$ is the thermal-activation-related mechanical threshold stress at 0 K, and $\Phi \left(\dot{\varepsilon },\;T\right)$ is the coefficient of influence related to strain rate and temperature (<1), indicating the role of thermal activation effects.

### 2.1. Thermal Stress

The thermal stress is closely related to the microstructural features of the material, as it utilizes the thermal vibrations of the crystal lattice to assist dislocations in overcoming weak barriers (such as forest dislocations and Peierls barriers). This facilitates the diffusion or glide of dislocations, resulting in material deformation.

#### 2.1.1. Thermal Activation Effect in Fixed Structures

During the thermal activation stage, the time required for dislocations to overcome barriers is primarily determined by the waiting time of $t_{w}$. In the mechanism of thermally activated dislocation motion, the average waiting time before encountering short-range barriers can be expressed in a modified form of the Arrhenius equation [22]:

(2) $$t_{w}=\frac{1}{\nu_{0}}\left[\exp\left(\frac{\Delta G}{k_{B}T}\right)-1\right]$$

where $\nu_{0}$ represents the vibration frequency (~10^11^ s^−1^) of the dislocation before encountering barriers [28], $k_{B}$ is the Boltzmann constant, *T* is the temperature, and ΔG is the thermal activation free energy (Gibbs free energy) required for dislocations to overcome the barriers, which is a function associated with the shape of the barrier (e.g., rectangular, square, triangular, parabolic, exponential) as well as stress. The modified form indicates that the waiting time for dislocations in a continuous slip system (ΔG=0) is zero.

Kocks and Ashby [28] proposed a general expression for activation energy:

(3) $$\Delta G=G_{0}{\left[1-{\left(\frac{\sigma_{th}}{{\hat{\sigma }}_{th}}\right)}^{p}\right]}^{q}$$

where $G_{0}$ is the reference free energy at 0 K ($=g_{0}\mu_{0}b^{3}$), $g_{0}$ is the normalized free energy, $\mu_{0}$ is the shear modulus ($=\mu \left(0K\right)$), and *b* is the magnitude of the Burgers vector. *p* and *q* are a pair of parameters that characterize the shape of the barrier. *q* corresponds to the shape of the short-range barrier, where for a quasi-parabolic barrier, the range is 1≤q≤2. *p* describes the rate of decay of the field at longer distances, with a value of 0<p≤1. A value of *p* = 1 indicates no decay, while smaller values indicate faster decay of the activation energy at longer distances.

According to Orowan’s law ($\gamma =N_{m}b\Delta l$), the equivalent true strain rate can be expressed as follows:

(4) $$\dot{\varepsilon }=\frac{N_{m}b\Delta l}{Mt_{w}}=\frac{N_{m}b\Delta l\nu_{0}}{M}{\left[\exp\left(\frac{\Delta G}{k_{B}T}\right)-1\right]}^{-1}$$

where Δl represents the average distance traveled by dislocations between barriers, and $N_{m}$ denotes the mobile dislocation density. In the high-strain-rate regime, the mobile dislocation density dominates the total dislocation density, that is $N_{m}\approx N_{\mathrm{tot}}$ [3,29]. *M* is the Taylor factor, where σ=Mτ, ε=γ/M, τ, and γ represent the shear stress and strain, respectively. The reference strain rate is defined as ${\dot{\varepsilon }}_{0}=N_{m}b\Delta l\nu_{0}/M$ with a general order of 10^7^ s^−1^ [13].

By substituting Equation (4) into Equation (3) and considering the relationship of $\Phi \left(\dot{\varepsilon },T\right)=\sigma_{\mathrm{th}}/{\hat{\sigma }}_{\mathrm{th}}$, the following expression is obtained:

(5) $$\Phi \left(\dot{\varepsilon }\right)={\left\{1-{\left[\frac{k_{B}T}{G_{0}}\ln\left(\frac{{\dot{\varepsilon }}_{0}}{\dot{\varepsilon }}+1\right)\right]}^{1/q}\right\}}^{1/p}$$

When the temperature exceeds the critical temperature $T_{\mathrm{cr}}$, the thermal activation stress term disappears, and the flow stress is solely determined by the athermal stress component, that is, $\sigma =\sigma_{\mathrm{ath}}$. The expression for the critical temperature is given by

(6) $$T_{\mathrm{cr}}={\left[\frac{k_{B}}{G_{0}}\ln\left(\frac{{\dot{\varepsilon }}_{0}}{\dot{\varepsilon }}+1\right)\right]}^{-1}$$

#### 2.1.2. Microstructural Evolution

Based on the analysis of microstructural evolution in deformation processing experiments, one of the key features of metal deformation is the variation of cell size with strain. Sevillano’s experimental results [30] demonstrate that the cell size decreases with increasing equivalent plastic strain. For certain metals, the ratio of the average cell size δ to its saturated size $\delta_{s}$ is a function of equivalent plastic strain, and the data points cluster around a curve that is independent of the metal type [31]. The normalized cell size $\tilde{\delta }$, defined as the ratio of the average cell size to its initial value $\delta_{0}$ ($\tilde{\delta }=\delta /\delta_{0}\leq 1$), can be described by the following evolution equation:

(7) $$\frac{d\tilde{\delta }}{d\varepsilon }=-\frac{\delta_{r}}{{\tilde{\delta }}_{s}}\left[{\tilde{\delta }}^{2}-{\tilde{\delta }}_{s}\tilde{\delta }\right]$$

where $\delta_{r}$ is a dimensionless coefficient that represents the rate of cell refinement, while ${\tilde{\delta }}_{s}$ denotes the normalized saturated cell size ($=\delta_{s}/\delta_{0}$). Both are functions related to strain rate and temperature. It is worth noting that this study is consistent with the theoretical framework proposed by Molinari and Ravichandran [25], which suggests that δ is a generalized characteristic length of the microstructure and does not have a direct relationship with geometric dimensions. Therefore, the use of the dimensionless size $\tilde{\delta }$ captures the essential trends in simulating microstructural evolution, where specific cell sizes of δ, $\delta_{0}$, and $\delta_{s}$ become less important, and there is no need to precisely capture quantitative results regarding the microstructure.

The integral Equation (7) yields the normalized cell size $\tilde{\delta }$, denoted as

(8) $$\tilde{\delta }=\frac{{\tilde{\delta }}_{s}}{1-\left(1-{\tilde{\delta }}_{s}\right)\exp\left(-\delta_{r}\varepsilon \right)}$$

The coefficients ${\tilde{\delta }}_{s}$ and $\delta_{r}$ can be expressed using the following empirical relationships [16]:

(9) $$\begin{array}{c} {\tilde{\delta }}_{s}={\tilde{\delta }}_{s0}\left[1-a_{s}{\left(\frac{\dot{\varepsilon }}{{\dot{\varepsilon }}_{r}}\right)}^{\xi_{s}}\times \max{\left(\frac{T}{T_{r}},1\right)}^{-\nu_{s}}\right]\\ \delta_{r}=\delta_{r0}\left[1+a_{r}{\left(\frac{\dot{\varepsilon }}{{\dot{\varepsilon }}_{r}}\right)}^{\xi_{r}}\times \max{\left(\frac{T}{T_{r}},1\right)}^{-\nu_{r}}\right] \end{array},\;(\dot{\varepsilon }<{\dot{\varepsilon }}_{r})$$

where $T_{r}$ and ${\dot{\varepsilon }}_{r}$ represent the reference temperature and strain rate, respectively. *a_s_* and *a_r_* are non-negative constants, and *ξ_s_*, *ν_s_*, *ξ_r_*, and *ν_r_* are constant values ranging from 0 to 1, controlling the dependence of ${\tilde{\delta }}_{s}$ and $\delta_{r}$ on strain rate and temperature. ${\tilde{\delta }}_{s0}$ represents the maximum reference saturated cell size $\delta_{s0}$ in comparison with its initial size (${\tilde{\delta }}_{s}\leq {\tilde{\delta }}_{s0}<1$), while $\delta_{r0}$ is the minimum reference refinement rate ($\delta_{r0}\leq \delta_{r}$). The normalized saturated cell size ${\tilde{\delta }}_{s}$ decreases with an increasing deformation rate and increases with an elevated temperature. Conversely, the cell refinement rate $\delta_{r}$ accelerates with higher deformation rates and decelerates with an increasing temperature. The evolution of these two parameters indicates that under low-temperature and high-strain-rate conditions, the dynamic cell refinement rate is faster, resulting in smaller achievable saturated sizes. In Equation (9), a modification is made to the temperature term, suggesting that the evolution of cell size becomes insensitive to temperature when it is lower than $T_{r}$.

Staker and Holt [32] proposed a simple relationship between the average cell size δ and the average total dislocation density $N_{\mathrm{tot}}$, which can be expressed as follows:

(10) $$\delta =\frac{K_{c}}{\sqrt{N_{\mathrm{tot}}}}$$

where *K*_c_ is a material constant, with a value of 16 for OFHC.

Therefore, the square root of the normalized dislocation density is inversely proportional to the normalized cell size $\tilde{\delta }$:

(11) $$\sqrt{\frac{N_{\mathrm{tot}}}{N_{0}}}=\frac{1}{\tilde{\delta }}$$

By substituting the saturation value into Equation (11), the normalized initial saturated cell size ${\tilde{\delta }}_{s0}=\sqrt{N_{0}/N_{s0}}$ can be obtained, where $N_{s0}$ represents the minimum reference value (~10^14^ m^−2^ [33]) of the saturated dislocation density.

#### 2.1.3. Threshold Stress and Thermal Stress

In the theory of MTS, the threshold stress associated with thermal activation is related to microstructural evolution [14] and can be expressed in combination with Equation (11) as

(12) $${\hat{\sigma }}_{th}=M\hat{\alpha }\mu \left(T\right)b\sqrt{N_{\mathrm{tot}}}=\frac{\mu \left(T\right)}{\mu_{0}}\frac{1}{\tilde{\delta }}\times \left(M\hat{\alpha }\mu_{0}b\sqrt{N_{0}}\right)$$

where $\hat{\alpha }$ represents the intensity of dislocation interaction (<1), and $\mu \left(T\right)$ is the temperature-dependent shear modulus. ${\hat{\sigma }}_{0}=M\hat{\alpha }\mu_{0}b\sqrt{N_{0}}$ represents a constant threshold stress value associated with the initial microstructure state at 0 K.

By substituting Equation (8) into Equation (12) and combining it with Equation (5), the thermal activation stress can be expressed as

(13) $$\sigma_{\mathrm{th}}=\frac{\;\tilde{\mu }}{{\tilde{\delta }}_{s}}{\hat{\sigma }}_{0}\left[1-\left(1-{\tilde{\delta }}_{s}\right)\exp\left(-\delta_{r}\varepsilon \right)\right]{\left\{1-{\left[\frac{k_{B}T}{G_{0}}\ln\left(\frac{{\dot{\varepsilon }}_{0}}{\dot{\varepsilon }}+1\right)\right]}^{1/q}\right\}}^{1/p}$$

here, $\tilde{\mu }$ is the normalized shear modulus ($=\mu \left(T\right)/\mu_{0}$). The thermal activation stress consists of two components: the structural evolution (threshold stress) part and the strain rate and temperature response of the material under a fixed structure. The structural evolution (normalized cell size) is related to strain rate and temperature. Therefore, the strain-rate sensitivity of the thermal activation stress can be divided into two parts: the rate sensitivity of material strain hardening caused by structural evolution and the instantaneous rate sensitivity of stress [15,34].

Equation (13) can also be written in the standard Voce–Kocks format [35]:

(14) $$\sigma_{\mathrm{th}}=\sigma_{\mathrm{th}}^{\varepsilon =\infty }-\left(\sigma_{\mathrm{th}}^{\varepsilon =\infty }-\sigma_{\mathrm{th}}^{\varepsilon =0}\right)\exp\left(-\delta_{r}\varepsilon \right)$$

where $\sigma_{\mathrm{th}}^{\varepsilon =\infty }$ and $\sigma_{\mathrm{th}}^{\varepsilon =0}$ represent the saturation and initial thermal activation stresses, respectively, with the following dependencies on strain rate and temperature:

(15) $$\sigma_{\mathrm{th}}^{\varepsilon =0}=\tilde{\mu }{\hat{\sigma }}_{0}\Phi \left(\dot{\varepsilon },T\right),\;\sigma_{\mathrm{th}}^{\varepsilon =\infty }=\sigma_{\mathrm{th}}^{\varepsilon =0}{\tilde{\delta }}_{s}^{-1}$$

### 2.2. Athermal Stress

The athermal stress is a result of the interaction between dislocations and long-range obstacles, known as the Hall–Petch strengthening effect [15]. The long-range obstacles mainly include thermally independent grain boundaries and far-field forest dislocations. As the grain size decreases (~μm), the number of grain boundaries increases, or the density of far-field forest dislocations increases with increasing deformation. These long-range obstacles hinder dislocation motion and capture more dislocations, thereby enhancing the material strength [36,37].

The Hall–Petch strengthening effect caused by the grain size effect can be expressed as follows [37,38]:

(16) $$\sigma_{G}=\sigma_{0}+k_{s}D_{0}^{-1/2}$$

where $\sigma_{0}$ represents the friction stress required for the motion of dislocations, which is typically negligible for FCC metals. $k_{s}$ is the strengthening coefficient, and *D*_0_ is the initial grain size.

The density of far-field forest dislocations $N_{f}$ can be calculated using the rate-independent dislocation density evolution equation proposed by Klepaczko [15], where the effective forest dislocation density ${\bar{N}}_{f}$ is represented as

(17) $${\bar{N}}_{f}=N_{f}-N_{f0}=\frac{M_{\mathrm{II}}}{k_{a0}}\left[1-\exp\left(-k_{a0}\varepsilon \right)\right]$$

where $M_{\mathrm{II}}$ and $k_{a0}$ are constants representing the proliferation rate and annihilation rate of dislocations, respectively. As the strain increases, the effective dislocation density approaches a saturation value, which is ${\bar{N}}_{\mathrm{fs}}=M_{\mathrm{II}}/k_{a0}$.

The athermal stress can be expressed as [1,29]

(18) $$\sigma_{\mathrm{ath}}=\sigma_{G}+\alpha_{d}\mu b\sqrt{{\bar{N}}_{f}}=\sigma_{0}+k_{s}D_{0}^{-1/2}+\;\tilde{\mu }B\sqrt{1-\exp\left(-k_{a0}\varepsilon \right)}$$

where *B* represents the stress at the saturation dislocation density at 0 K, denoted as $B=\alpha_{d}\mu_{0}b\sqrt{{\bar{N}}_{\mathrm{fs}}}$, and $\alpha_{d}$ is a material constant (<1). The athermal stress indirectly achieves the thermal softening effect of the material through the relationship between the shear modulus (elastic field) and temperature. It realizes strain hardening of the material through the generation and storage of dislocations during deformation, while the deformation rate does not affect the evolution of nonthermal stress.

### 2.3. Flow Stress and Hardening Rate

Finally, combining Equations (13) and (18), the constitutive relationship for the flow stress of FCC metals is obtained as

(19) $$\begin{array}{l} \sigma =\sigma_{0}+k_{s}D_{0}^{-1/2}+\;\tilde{\mu }B\sqrt{1-\exp\left(-k_{a0}\varepsilon \right)}\\ +\;\tilde{\mu }\frac{{\hat{\sigma }}_{0}}{{\tilde{\delta }}_{s}}\left[1-\left(1-{\tilde{\delta }}_{s}\right)\exp\left(-\delta_{r}\varepsilon \right)\right]{\left\{1-{\left[\frac{k_{B}T}{G_{0}}\ln\left(\frac{{\dot{\varepsilon }}_{0}}{\dot{\varepsilon }}+1\right)\right]}^{1/q}\right\}}^{1/p} \end{array}$$

The strain hardening rate with respect to stress is given by

(20) $$\frac{d\sigma }{d\varepsilon }=\frac{d\sigma_{\mathrm{ath}}}{d\varepsilon }+\frac{d\sigma_{\mathrm{th}}}{d\varepsilon }=\frac{k_{a0}}{2}\left[\frac{{\left(\sigma_{\mathrm{ath}}^{\varepsilon =\infty }-\sigma_{G}\right)}^{2}}{\sigma_{\mathrm{ath}}-\sigma_{G}}-\left(\sigma_{\mathrm{ath}}-\sigma_{G}\right)\right]+\delta_{r}\left(\sigma_{\mathrm{th}}^{\varepsilon =\infty }-\sigma_{\mathrm{th}}\right)$$

where the first term represents the strain hardening rate induced by the evolution of long-range obstacles in the microstructure, such as grain size and grain boundaries. The hardening rate initially decreases inversely proportional to the athermal stress and follows a linear function with a constant slope $k_{a0}$ at higher stresses. The second term accounts for the strain hardening rate due to thermal stress arising from the evolution of short-range obstacles, such as line defects represented by dislocation spacing. Under stable loading conditions, the hardening rate decreases linearly as the thermal stress increases, where $\delta_{r}$ is a constant.

Observing the model’s behavior under constant loading conditions (stable strain rate and temperature), the overall strain hardening rate exhibits a rapid decrease as the deformation intensifies due to the inverse proportional function, reflecting the hardening characteristics of FCC metals at stage III [14,31]. Once the material reaches a certain level of strengthening, the overall strain hardening rate gradually decreases linearly until it approaches zero, with a slope approximately equal to $-\left(k_{a0}+\delta_{r}\right)$. This behavior is consistent with the hardening characteristics of stage IV [31,39]. Since $k_{a0}$ is a material constant, the sensitivity of the hardening rate to strain rate and temperature primarily depends on the microstructural refinement rate $\delta_{r}$.

### 2.4. Shear Modulus

The Nadal-Le Poac (NP) shear modulus model [40] replaces the empirical temperature dependence of the shear modulus in the SCG model with an equation based on the Lindemann melting theory. This modification enhances the robustness of the model when applied under high-temperature and high-pressure conditions. Moreover, the NP model mitigates the instantaneous decrease of the shear modulus at the melting point. The zero-pressure form of the NP shear modulus model can be expressed as

(21) $$\begin{array}{l} \mu \left(T\right)=\frac{1}{\Gamma \left(T/T_{m}\right)}\left[\mu_{0}\left(1-\frac{T}{T_{m}}\right)+\frac{\rho_{m}}{\tilde{C}m_{u}}k_{B}T\right]\\ \Gamma \left(T/T_{m}\right)=1+\exp\left[-\frac{1+1/\zeta_{m}}{1+\zeta_{m}/\left(1-T/T_{m}\right)}\right] \end{array}$$

where $\rho_{m}$ represents the material’s standard density, *T_m_* denotes the melting point of the material under standard conditions, $\tilde{C}$ is a material constant related to the Lindemann constant, $m_{u}$ signifies the atomic mass, $\Gamma \left(T/T_{m}\right)$ controls its nonlinear behavior when $T/T_{m}\approx 1$, and $\zeta_{m}\ll 1$ serves as a fitting parameter that governs the overall fit quality.

### 2.5. Adiabatic Temperature Rise

During the metal deformation process, it was experimentally found that the majority of the energy supplied to effect plastic deformation converted to heat, contributing to an increase in temperature [41,42]. The conversion coefficient *β* generally falls within the range of 0.9 to 1.0 for high deformation rates (>1 s^−1^) in adiabatic processes [42,43]. If adiabatic shear banding occurs under high strain rates, the heat generated during plastic deformation can lead to a rapid increase in temperature within the localized region of the adiabatic shear band. The equation connecting temperature raising and plastic work is presented in an integral format [44]:

(22) $$T=T_{0}+\frac{\beta }{\rho_{m}C_{p}}\int_{0}^{\varepsilon }\sigma \left(\varepsilon ,\dot{\varepsilon },T\right)d\varepsilon$$

where *T*_0_ is the initial temperature, and *C_p_* is the specific heat of the material at constant pressure. In the calculation of flow stress during adiabatic processes, it is necessary to update the temperature state variable at each incremental strain step.

## 3. Application

This section presents the material parameters used in the model for annealed OFHC and compares the predicted results with experimental data. The results cover the influence of strain rate on flow stress under fixed strains, as well as the deformation behavior of annealed OFHC across a wide range of strain rates (4 × 10^−4^ to 6.4 × 10^5^ s^−1^) at room temperature, and a broad temperature range (77 to 1096 K) at a strain rate of 4000 s^−1^.

### 3.1. Parameter Calibration

The physical constants of annealed OFHC [43] and the parameters in the NP shear modulus model at zero hydrostatic pressure [40] are shown in Table 1.

In the component of athermal stress, Meyers et al. [37] conducted a study and found that the value of *k_s_* for OFHC decreases linearly with increasing strain under low strain rates, ranging from 56 to 260 MPa$\sqrt{\mu m}$. However, at high strain rates, the value remains approximately constant. This behavior is taken into account in the ZA model [19], where an intermediate value is employed. The coefficients for the athermal stress in the model are provided in Table 2.

The material parameters for the thermal stress component are described in the following two tables. Table 3 provides the parameters that are independent of microstructural evolution, calculated  σ ^0 as 50.2 MPa based on the data.

The parameters for the normalized cell size evolution model are proposed by Molinari and Ravichandran [25] and illustrated in Table 4. By utilizing the data in the table and Equation (11), the minimum value of the reference saturated dislocation density is approximately 7 × 10^14^ m^−2^, which consistent with the actual physical laws.

### 3.2. Simulation Results

**Figure 1:**
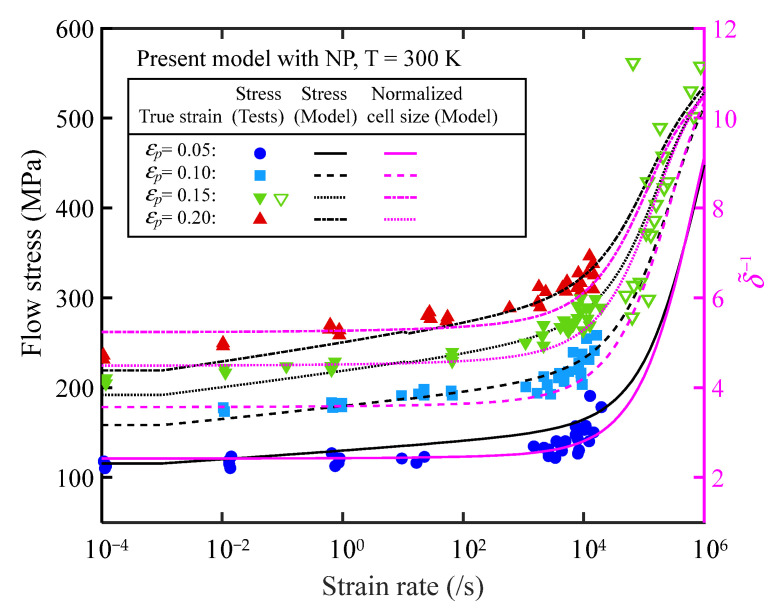
Relationship between flow stress, normalized cell size, and strain rate of annealed OFHC at fixed strain and room temperature.

The experimental values for a comparison with the model calculations can be found in Appendix A. In Figure 1, the relationships between flow stress, normalized cell size, and strain rate for OFHC at fixed strains and room temperature are depicted. It is noteworthy that the sensitivity of flow stress to strain rate undergoes a sudden change at 10^4^ s^−1^, which aligns with the evolution pattern of the normalized reciprocal cell size ${\tilde{\delta }}^{-1}$. This observation suggests that the sensitivity of flow stress to strain rate is dominated by the microstructural evolution law. Specifically, when $\dot{\varepsilon }$ < 10^4^ s^−1^, the normalized reciprocal cell size lines are horizontally parallel with fixed strains. This behavior arises from the fact that the saturation size and refinement rate, as controlled by Equation (9), remain approximately constant, indicating that the microstructure exhibits low sensitivity to changes in strain rate. Additionally, the sensitivity of flow stress to strain rate is influenced by the term $\Phi \left(\dot{\varepsilon },T\right)$. At extremely low strain rates ($\dot{\varepsilon }$ < 10^−3^ s^−1^), the value of $\Phi \left(\dot{\varepsilon },T\right)$ exhibits minimal variation with strain rate, implying that the flow stress is predominantly determined by the deformation level and temperature.

**Figure 2:**
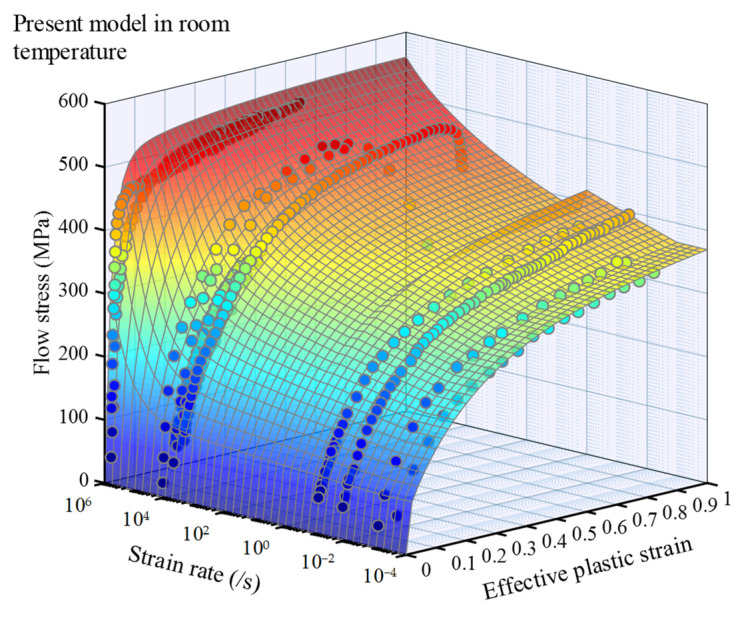
Flow stress–strain curved surface of annealed OFHC over a wide strain rate range at room temperature, where the dots are experimental data referred in Table A2, with blue indicating low stress and red indicating high stress.

**Figure 3:**
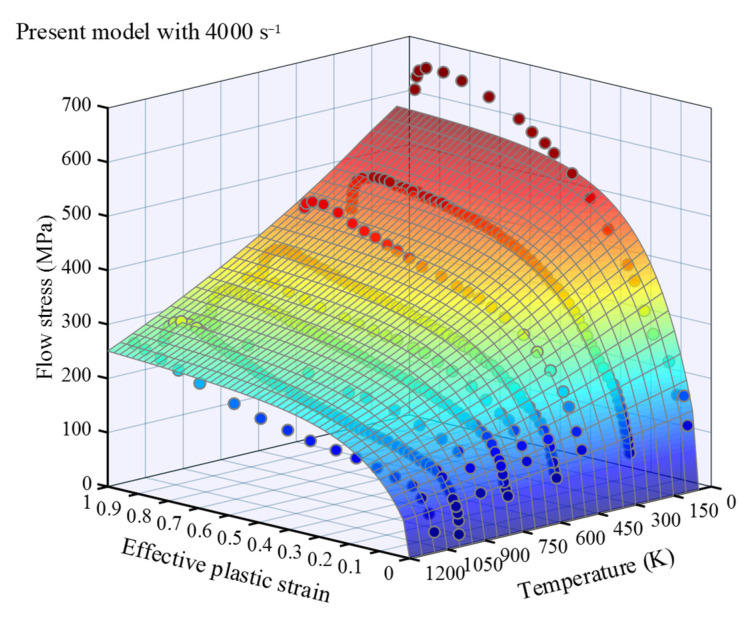
Flow stress–strain curved surface of annealed OFHC over a wide temperature range with $\dot{\varepsilon }=$4000 s^−1^, where the dots are experimental data referred in Table A3, with blue indicating low stress and red indicating high stress.

Figure 2 and Figure 3 present the relationship between flow stress and strain within a wide range of strain rates at room temperature and a wide range of temperatures at a fixed strain rate for OFHC, respectively. The results demonstrate a strong agreement between the model’s predicted values and the experimental data, which will be further analyzed in the subsequent section to quantify the error. In Figure 2, when the strain exceeds $\varepsilon_{1}$ (≈0.2), a distinct change in the surface occurs near 1 s^−1^ for a fixed strain. Within a narrow range of strain rates, an intriguing phenomenon emerges as the flow stress decreases with an increasing strain rate. This intriguing behavior can be attributed to the consideration of temperature increase induced by plastic deformation when $\dot{\varepsilon }$ > 1 s^−1^, where the thermal softening effect of the material surpasses the strengthening effect associated with strain rate.

## 4. Result Analysis and Discussion

### 4.1. Relative Error Analysis

Based on the experimental results of OFHC across a wide range of strain rates and deformation temperatures, Biswajit Banerjee conducted a meticulous evaluation in his research [47,48]. He identified the hierarchical order of the model’s evaluation errors: PTW, MTS, ZA, JC, and SCGL (Steinberg–Cochran–Guinan–Lund [49]). Notably, the present model’s microstructure evolution is predicated upon the MR model, while the validation experiments predominantly stem from Nemat-Nasser and Li [20]. To enhance the understanding of the predictive accuracy of the constitutive models, the paper undertook a thorough analysis of errors and evaluated the performance of not only the present model but also five alternative constitutive models: MTS, PTW, JC, NNL, and MR. This comprehensive assessment encompassed a total of six distinct models, providing valuable insights into their respective prediction capabilities.

The relative error values $E_{i,j}$ are computed using Equation (23):

(23) $$E_{i,j}\left(\%\right)=\frac{\sigma_{i,j}^{\mathrm{pre}}-\sigma_{i,j}^{\exp}}{\sigma_{i,j}^{\exp}}\times 100\%$$

where $\sigma_{i,j}^{\mathrm{pre}}$ denotes the model-predicted flow stress and $\sigma_{i,j}^{\exp}$ represents the experimental flow stress. The subscripts ‘*i*’ and ‘*j*’ signify the relative error for the *i*-th data point under the *j*-th deformation condition.

**Figure 4:**
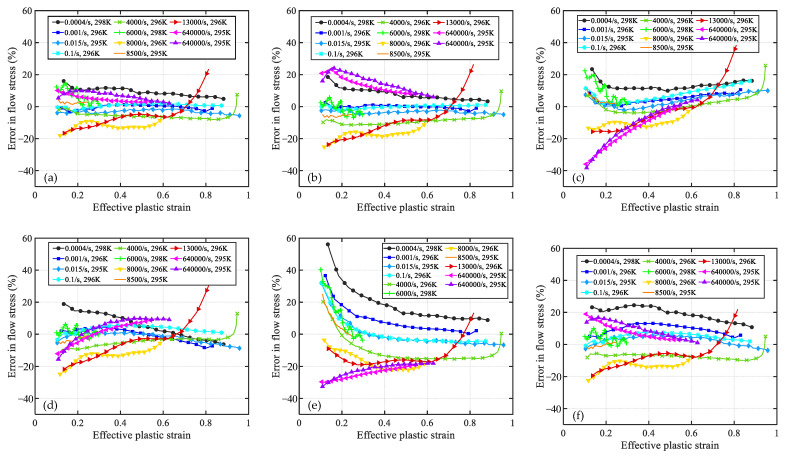
The relative error curve between the predicted values of models and the experimental results over a wide range of strain rates at room temperature. The models are (**a**) Present, (**b**) MTS, (**c**) NNL, (**d**) PTW, (**e**) JC, and (**f**) MR.

**Figure 5:**
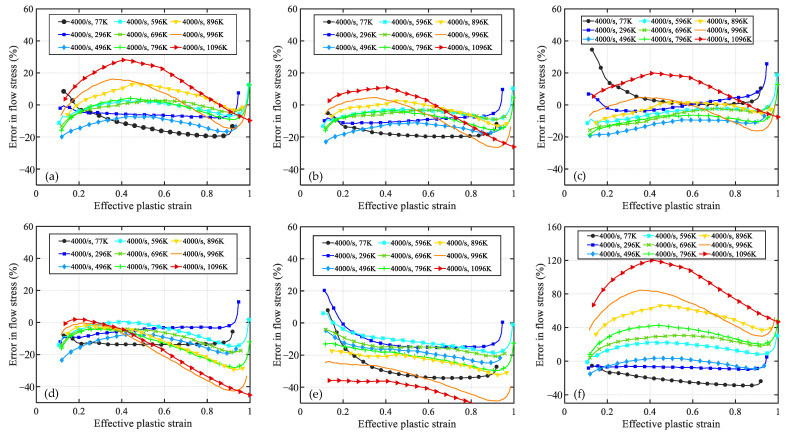
The relative error curve between the predicted values of models and the experimental results over a wide range of temperatures at a strain rate of 4000 s^−1^. The models are (**a**) Present, (**b**) MTS, (**c**) NNL, (**d**) PTW, (**e**) JC, and (**f**) MR.

Figure 4 and Figure 5 showcase the error curves representing the disparities between the predicted flow stress of OFHC using six distinct models and the corresponding experimental results. The order in which these models are presented is as follows: (a) present, (b) MTS, (c) NNL, (d) PTW, (e) JC, and (d) MR. Models (a) to (d) encompass constitutive models that incorporate microstructure evolution, while model (e) is an empirical model and (f) is a semiempirical physical model.

As shown in Figure 4a, the present model exhibits relatively larger relative errors (at ±20%) in predicting results at room temperature and strain rates of 8000 s^−1^ and 1.3 × 10^4^ s^−1^. However, under other strain-rate conditions, the relative errors of the model are mainly within ±10%. Notably, the relative error curves of the other five models at the above two strain rates exhibit similar trends to that of the present model. Therefore, it is plausible that these significant discrepancies may stem from experimental errors at these two strain rates. Regarding extremely high strain rates (6.4 × 10^5^ s^−1^), the present model, PTW, and MR demonstrate relatively accurate prediction results, while the MTS model exhibits slightly higher values compared with the present. Both the NNL and JC models significantly underestimate the experimental results at low strains, with relative errors approaching −40%. Nevertheless, the accuracy of NNL predictions notably improves as the strain increases, while the JC model remains approximately −20%. Moreover, under all other strain-rate conditions, the JC model overestimates material flow stress at low strains with relative errors exceeding +20%. Excluding the JC model, the other models exhibit satisfactory consistency with the experimental results across the range of 0.001–8500 s^−1^. However, at an extremely low strain rate of 4 × 10^−4^ s^−1^, all models predict higher values than the experimental results. Generally speaking, the predictions of the present model outperform those of the other models. This discrepancy can be attributed to the fact that the dislocation slip velocity is slower at relatively low strain rates, resulting in prolonged interaction time between dislocations and internal defects or barriers within the material. Consequently, dislocation diffusion or dispersion mechanisms may become the dominant deformation mechanisms, leading to a decrease in material strength and causing the model to predict higher flow stress values compared with the experimental data [19,50].

The relative error curves of Figure 5a,b,f exhibit a high degree of similarity. These error curves demonstrate a consistent pattern, where the curves gradually shift upwards as the temperature increases, indicating that the predicted values are higher compared with the experimental results at high temperatures. Furthermore, for model (c) NNL, its predicted values are consistently higher than the experimental values at both high and low temperatures. This behavior can be attributed to the fact that, under high temperature, the solid-state diffusion rate in the crystal significantly increases, promoting grain growth. Larger grains may contain more structural defects or dislocations, resulting in a decrease in material strength. Since these models do not account for microstructural coarsening, the predicted results at high temperatures tend to be higher than the experimental ones. On the other hand, the PTW and JC models underestimate the material’s flow stress at higher temperatures, and as the strain increases, their predictions deviate even further from the experimental results. From the perspective of the error curves, the MTS model exhibits the best predictive performance, while there is still room for improvement in the present model’s predictions at higher temperatures (>0.7 *T*_m_).

### 4.2. Comprehensive Error Analysis

To provide a more comprehensive assessment of the predictive capabilities of the abovementioned six constitutive models for the flow stress of OFHC, three statistical average errors were employed to evaluate the model predictions. These include the mean absolute relative error of plastic work (MARE-P.WK, ${\bar{E}}_{\mathrm{AR}.W}$), the mean absolute relative error of flow stress (MARE-F.S, ${\bar{E}}_{\mathrm{AR}.Y}$), and the mean maximum absolute error of flow stress (MMAE-F.S, ${\bar{E}}_{\mathrm{MA}.Y}$). The calculation formulas for these metrics are as follows:

(24) $${\bar{E}}_{\mathrm{AR}.W}=\frac{1}{J}\sum_{j=1}^{J}\frac{W_{j}^{\mathrm{pre}}-W_{j}^{\exp}}{W_{j}^{\exp}}\times 100\%,\;W_{j}=\int_{0}^{\varepsilon }\sigma_{j}d\varepsilon$$

(25) $${\bar{E}}_{\mathrm{AR}.Y}=\frac{1}{J}\sum_{j=1}^{J}\left(\frac{1}{I_{j}}\sum_{i=1}^{I_{j}}\left|E_{i,j}\right|\right)$$

(26) $${\bar{E}}_{\mathrm{MA}.Y}=\frac{1}{J}\sum_{j=1}^{J}\left(\left|{\bar{E}}_{j}\right|+\sqrt{\frac{1}{I_{j}}\sum_{i=1}^{I_{j}}{\left(E_{i,j}-{\bar{E}}_{j}\right)}^{2}}\right),\;{\bar{E}}_{j}=\frac{1}{I_{j}}\sum_{i=1}^{I_{j}}E_{i,j}$$

where $I_{j}$ represents the total number of data points with the deformation condition of index *j*, ${\bar{E}}_{j}$ denotes the average value of the relative error curve *j*, and *J* refers to the total number of experimental data that satisfy the statistical criteria. For example, there are four sets of experimental data with $\dot{\varepsilon }$ ≤ 10^3^ s^−1^ at room temperature, that is, *J* = 4.

**Figure 6:**
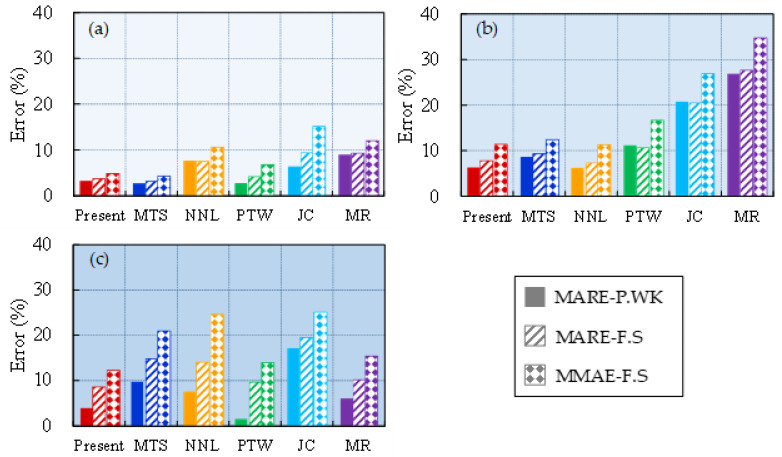
Three statistical average errors of model predictions within (**a**) $\dot{\varepsilon }\leq 10^{3}s^{-1}$, (**b**) $10^{3}s^{-1}<\;\dot{\varepsilon }\leq 10^{4}s^{-1}$, and (**c**) $\dot{\varepsilon }\;>10^{4}s^{-1}$: MARE-P.WK, MARE-F.S, and MMAE-F.S.

The statistical average errors for the predictions of the six models under different strain-rate gradients at room temperature are presented in Figure 6. It is observed that the majority of the models perform well in accurately capturing the flow stress behavior of the material at low strain rates, with the exception of the JC model, which exhibits a higher MMAE-F.S value. The strain-rate sensitivity of the flow stress undergoes a notable transition within the range of 10^3^ s^−1^–10^4^ s^−1^. Specifically, the present model and the MTS and NNL models exhibit relatively low statistical average errors of approximately 10% within this range, while the JC and MR models demonstrate larger errors exceeding >20%. This finding indicates that the abrupt change in flow stress sensitivity of OFHC is significantly influenced by microstructural evolution. Moreover, at extremely high strain rates, the values of MMAE-F.S for the MTS, NNL, and JC models exceed 20%, suggesting that these three models may not yield accurate results under such extreme high-strain-rate conditions.

**Figure 7:**
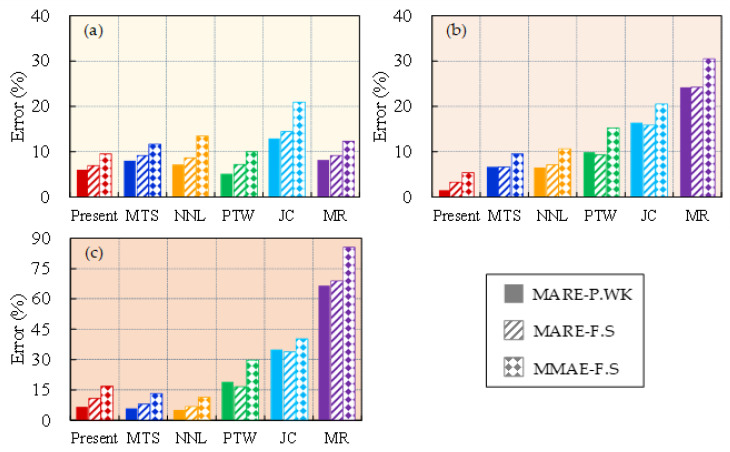
Three statistical average errors of model predictions within (**a**) *T* ≤ 0.4 *T*_m_, (**b**) 0.4 *T*_m_ < *T* ≤ 0.6 *T*_m_, and (**c**) *T* > 0.6 *T*_m_: MARE-P.WK, MARE-F.S, and MMAE-F.S.

The statistical average errors for the predictions of the six models under different temperature gradients at a strain rate of 4000 s^−1^ are shown in Figure 7. It is observed that the present model and the MTS and NNL models consistently demonstrate relatively small statistical average errors across all temperature ranges (maximum value is approximately 10%). The PTW and MR models demonstrate statistical average errors below 10% at temperatures below 0.4 *T*_m_. However, as the temperature increases, the statistical average errors increase as well. Notably, the MR model exhibits statistical average errors exceeding 20% within the intermediate temperature range and even surpasses 60% at high temperatures. Therefore, the MR and PTW models are deemed unsuitable for high-temperature conditions. Moreover, throughout the entire temperature range, the JC model displays statistical average errors ranging from 15% to 30%. This indicates that the standard JC model is not suitable for simulations involving strain rates equal to or greater than 4000 s^−1^. The JC model, being an empirical model that is based on experimental test data for parameter fitting, lacks a robust physical foundation. The information should only be utilized within the specified parameters for accurate results and should not be applied to conditions outside of those parameters. Otherwise, it may result in substantial errors and inaccurate predictions of material behavior.

**Figure 8:**
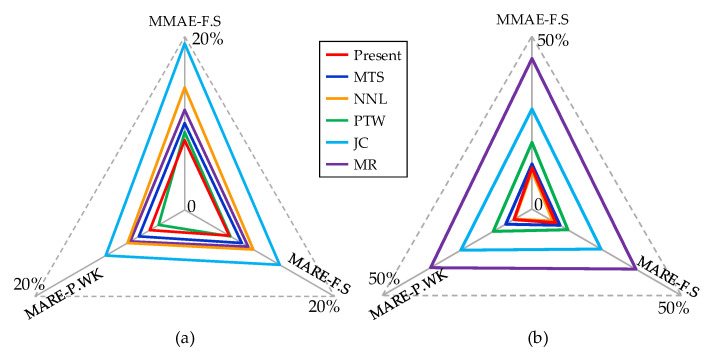
Comprehensive evaluation of three statistical average errors in model prediction results: (**a**) in different strain rates and (**b**) in different temperatures.

The statistical average errors in model predictions across all strain rates and temperature ranges are comprehensively evaluated and presented in Figure 8. The enclosed triangle area formed by the lines serves as a measure of the model’s prediction accuracy, with a smaller area indicating higher precision. When considering different strain rates, the sizes of the triangles formed by the three statistical average errors follow this order: present ≈ PTW < MTS < MR < NNL < JC. Similarly, for varying temperatures, the triangle sizes adhere to this sequence: present ≈ NNL < MTS < PTW < JC < MR. From the results, it is evident that the proposed model demonstrates favorable simulation outcomes across a broad range of strain rates and temperatures.

## 5. Conclusions

This study develops a semiempirical physical constitutive model based on a normalized cell size evolution model, along with the dislocation density evolution mechanism and the impact of dislocation motion on plastic deformation. This single and continuous model, which captures the sensitivity of microstructure evolution to strain rates, is capable of effectively capturing the upturning in flow stress at strain rates exceeding the critical value (~10^4^ s^−1^) at a fixed strain. Furthermore, it demonstrates remarkable precision across a wide range of strain rates (4 × 10^−4^–6.4 × 10^5^ s^−1^) and temperatures (77–1096 K). Despite this, the model is not without its limitations. The high-temperature environment presents challenges in the nonlinear mechanical behavior of materials, such as grain growth and structural defect generation. The ramifications of these phenomena on the plastic deformation process and the impact of temperature on microstructural evolution warrant further examination.

Upon comparison of the predictive capabilities of the present model with those of the MTS, NNL, PTW, JC, and MR models, it was determined that the microstructure-based constitutive model, particularly the present model, offers a more accurate representation of material plastic deformation behavior under high strain rates. The proposed model surpasses others in terms of its predictive accuracy. The results of this investigation hold a significant value as they provide guidance for selecting appropriate models for engineering applications and augmenting our understanding of the rheological behavior of OFHC.

## Figures and Tables

**Table 1:** Parameters in the NP shear modulus model and temperature evolution model of OFHC.

*ρ*_m_ (kg/m^3^)	*C_p_* (J/kg/K)	*β*	*μ*_0_ (GPa)	*T*_m_ (K)	*ζ*_m_	$\tilde{C}$	*m_u_* (amu)
8930	382	0.9	50.7	1356	0.04	0.057	63.55

**Table 2:** Material coefficients in the athermal stress component.

$\sigma_{G}$(MPa)	$k_{s}\;(\mathrm{MPa}\sqrt{\mu m})$	*D*_0_ (μm)	*B* (MPa)	$k_{a0}$
20.6	158 [19]	62	230 [3]	0.64 [3]

**Table 3:** Material parameters in the thermal stress component that are independent of microstructure.

*N*_0_ (m^−2^)	*M*	$\hat{\alpha }$	*b* (nm)	$k_{B}/G_{0}$ (K^−1^)	*p*	*q*	${\dot{\varepsilon }}_{0}$ (s^−1^)
10^13^ [44]	3.06 [45]	0.4 [46]	0.256 [31]	4.9 × 10^−5^ [20]	2/3 [13]	1 [13]	10^7^ [13]

**Table 4:** Parameters of the evolution equation for normalized cell size [25].

${\tilde{\delta }}_{s0}$	$a_{s}$	$\xi_{s}$	$\nu_{s}$	$\delta_{r0}$	$a_{r}$	$\xi_{r}$	$\nu_{r}$	${\dot{\varepsilon }}_{r}$ (s^−1^)	$T_{r}$ (K)
0.12	0.377	0.24	0.5	4.3	50	0.8	0	10^7^	298

## Data Availability

The data referenced can be found in Appendix A.

## Appendix A. Source of Experimental Data

**Table A1:** Experimental results on the relationship between flow stress and strain rate of OFHC at fixed strain and room temperature.

Condition	ε	$\dot{\varepsilon }$ (s^−1^)	*T* (K)	References	Purity (%)
Fixed strain and room temperature	0.15	$\dot{\varepsilon }\in$6 × (10^4^, 10^5^)	300	Tong and Clifton (1992) [51]	99.99
	0.05, 0.10, 0.15, 0.20	$\dot{\varepsilon }\in$(10^−4^, 2 × 10^4^)		Preston, Tonks, and Wallace (2003) [24]	99.99

**Table A2:** Experimental results on the flow stress–strain relationship of OFHC over a wide range of strain rates at room temperature.

Condition	$\dot{\varepsilon }$ (s^−1^)	*T* (K)	References	Purity (%)
Room temperature and wide range of strain rates	0.015, 8500, 6.4 × 10^5^	295	Follansbee and Kocks (1988) [13]	99.99
	0.001, 0.1, 4000, 8000	296	Nemat-Nasser and Li (1998) [20]	99.98
	0.0004, 6000	298	Tanner and McGinty (1999) [52]	99.99
	1.3 × 10^4^	296	Jing and Wang (2022) [53]	Not mentioned

**Table A3:** Experimental results on the flow stress–strain relationship of OFHC over a wide range of temperatures at a fixed strain rate.

Condition	$\dot{\varepsilon }$	*T* (K)	References	Purity (%)
Fixed strain rate and wide range of temperatures	4000	77, 296, 496, 596, 696, 796, 896, 996, 1096	Nemat-Nasser and Li (1998) [20]	99.98

## References

[1] Salvado F., Teixeira-Dias F., Walley S.M., Lea L., Cardoso J. (2017). A Review on the Strain Rate Dependency of the Dynamic Viscoplastic Response of FCC Metals. Prog. Mater. Sci..

[2] Armstrong R.W., Walley S.M. (2008). High Strain Rate Properties of Metals and Alloys. Int. Mater. Rev..

[3] Gao C.Y., Zhang L.C. (2012). Constitutive Modelling of Plasticity of Fcc Metals under Extremely High Strain Rates. Int. J. Plast..

[4] Jia X., Hao K., Luo Z., Fan Z. (2022). Plastic Deformation Behavior of Metal Materials: A Review of Constitutive Models. Metals.

[5] Johnson G.R., Cook W.H. A Constitutive Model and Data for Materials Subjected to Large Strains. Proceedings of the 7th International Symposium on Ballistics.

[6] Malvern L.E. (1951). The Propagation of Longitudinal Waves of Plastic Deformation in a Bar of Material Exhibiting a Strain-Rate Effect. J. Appl. Mech..

[7] Perzyna P., Chernyi G.G., Dryden H.L., Germain P., Howarth L., Olszak W., Prager W., Probstein R.F., Ziegler H. (1966). Fundamental Problems in Viscoplasticity. Advances in Applied Mechanics.

[8] Campbell J.D. (1973). Dynamic Plasticity: Macroscopic and Microscopic Aspects. Mater. Sci. Eng..

[9] Rule W.K., Jones S.E. (1998). A Revised Form for the Johnson–Cook Strength Model. Int. J. Impact Eng..

[10] Wang J., Guo W.-G., Li P., Zhou P. (2017). Modified Johnson-Cook Description of Wide Temperature and Strain Rate Measurements Made on a Nickel-Base Superalloy. Mater. High Temp..

[11] Wang Y., Zeng X., Chen H., Yang X., Wang F., Zeng L. (2021). Modified Johnson-Cook Constitutive Model of Metallic Materials under a Wide Range of Temperatures and Strain Rates. Results Phys..

[12] Kumar Reddy Sirigiri V., Yadav Gudiga V., Shankar Gattu U., Suneesh G., Mohan Buddaraju K. (2022). A Review on Johnson Cook Material Model. Mater. Today Proc..

[13] Follansbee P.S., Kocks U.F. (1988). A Constitutive Description of the Deformation of Copper Based on the Use of the Mechanical Threshold Stress as an Internal State Variable. Acta Metall..

[14] Mecking H., Kocks U.F. (1981). Kinetics of Flow and Strain-Hardening. Acta Metall..

[15] Klepaczko J.R. (1991). Physical-State Variables—The Key to Constitutive Modeling in Dynamic Plasticity. Nucl. Eng. Des..

[16] Mohamadnejad S., Basti A., Ansari R. (2020). Analyses of Dislocation Effects on Plastic Deformation. Multiscale Sci. Eng..

[17] Lea L.J., Jardine A.P. (2018). Characterisation of High Rate Plasticity in the Uniaxial Deformation of High Purity Copper at Elevated Temperatures. Int. J. Plast..

[18] Lea L., Brown L., Jardine A. (2020). Time Limited Self-Organised Criticality in the High Rate Deformation of Face Centred Cubic Metals. Commun. Mater..

[19] Zerilli F.J., Armstrong R.W. (1987). Dislocation-mechanics-based Constitutive Relations for Material Dynamics Calculations. J. Appl. Phys..

[20] Nemat-Nasser S., Li Y. (1998). Flow Stress of fcc Polycrystals with Application to OFHC Cu. Acta Mater..

[21] Gao C.Y., Zhang L.C. (2010). A Constitutive Model for Dynamic Plasticity of FCC Metals. Mater. Sci. Eng. A.

[22] Austin R.A., McDowell D. (2011). A Dislocation-Based Constitutive Model for Viscoplastic Deformation of Fcc Metals at Very High Strain Rates. Int. J. Plast..

[23] Austin R.A., McDowell D.L. (2012). Parameterization of a Rate-Dependent Model of Shock-Induced Plasticity for Copper, Nickel, and Aluminum. Int. J. Plast..

[24] Preston D., Tonks D., Wallace D. (2003). Model of Plastic Deformation for Extreme Loading Conditions. J. Appl. Phys..

[25] Molinari A., Ravichandran G. (2005). Constitutive Modeling of High-Strain-Rate Deformation in Metals Based on the Evolution of an Effective Microstructural Length. Mech. Mater..

[26] Durrenberger L., Molinari A., Rusinek A. (2008). Internal Variable Modeling of the High Strain-Rate Behavior of Metals with Applications to Multiphase Steels. Mater. Sci. Eng. A.

[27] Durrenberger L., Molinari A. (2009). Modeling of Temperature and Strain-Rate Effects in Metals Using an Internal Variable Model. Exp. Mech..

[28] Kocks U.F., Argon A.S., Ashby M.F. (1975). Thermodynamics and Kinetics of Slip. Prog. Mater. Sci..

[29] Wang Z.Q., Beyerlein I.J., LeSar R. (2008). Slip Band Formation and Mobile Dislocation Density Generation in High Rate Deformation of Single Fcc Crystals. Philos. Mag..

[30] Gil Sevillano J., van Houtte P., Aernoudt E. (1980). Large Strain Work Hardening and Textures. Prog. Mater. Sci..

[31] Nes E. (1997). Modelling of Work Hardening and Stress Saturation in FCC Metals. Prog. Mater. Sci..

[32] Staker M.R., Holt D.L. (1972). The Dislocation Cell Size and Dislocation Density in Copper Deformed at Temperatures between 25 and 700 °C. Acta Metall..

[33] Estrin Y., Kubin L.P. (1986). Local Strain Hardening and Nonuniformity of Plastic Deformation. Acta Metall..

[34] Voce E. (1948). The Relationship between Stress and Strain for Homogeneous Deformations. J. Inst. Met..

[35] Hall E.O. (1951). The Deformation and Ageing of Mild Steel: III Discussion of Results. Proc. Phys. Soc. Sect. B.

[36] Armstrong R.W. (1987). The (Cleavage) Strength of Pre-Cracked Polycrystals. Eng. Fract. Mech..

[37] Meyers M.A., Andrade U.R., Chokshi A.H. (1995). The Effect of Grain Size on the High-Strain, High-Strain-Rate Behavior of Copper. Met. Mater. Trans. A.

[38] Yan S., Yang H., Li H., Yao X. (2016). A Unified Model for Coupling Constitutive Behavior and Micro-Defects Evolution of Aluminum Alloys under High-Strain-Rate Deformation. Int. J. Plast..

[39] Kocks F., Mecking H. (2003). Physics and Phenomenology of Strain Hardening: The FCC Case. Prog. Mater. Sci..

[40] Nadal M.-H., Le Poac P. (2003). Continuous Model for the Shear Modulus as a Function of Pressure and Temperature up to the Melting Point: Analysis and Ultrasonic Validation. J. Appl. Phys..

[41] Klepaczko J., Rusinek A., Rodríguez-Martínez J.A., Pęcherski R., Arias A. (2009). Modelling of Thermo-Viscoplastic Behaviour of DH36 and Weldox 460-E Structural Steels at Wide Ranges of Strain Rates and Temperatures, Comparison of Constitutive Relations for Impact Problems. Mech. Mater..

[42] Baig M., Khan A.S., Choi S.-H., Jeong A. (2013). Shear and Multiaxial Responses of Oxygen Free High Conductivity (OFHC) Copper over Wide Range of Strain-Rates and Temperatures and Constitutive Modeling. Int. J. Plast..

[43] Mason J.J., Rosakis A.J., Ravichandran G. (1994). On the Strain and Strain Rate Dependence of the Fraction of Plastic Work Converted to Heat: An Experimental Study Using High Speed Infrared Detectors and the Kolsky Bar. Mech. Mater..

[44] Voyiadjis G.Z., Abed F.H. (2005). Effect of Dislocation Density Evolution on the Thermomechanical Response of Metals with Different Crystal Structures at Low and High Strain Rates and Temperatures. Arch. Mech..

[45] Humphreys F.J., Hatherly M. (2004). Recrystallization and Related Annealing Phenomena.

[46] Huang M., Rivera-Díaz-del-Castillo P.E.J., Bouaziz O., van der Zwaag S. (2009). A Constitutive Model for High Strain Rate Deformation in FCC Metals Based on Irreversible Thermodynamics. Mech. Mater..

[47] Banerjee B. (2005). Taylor Impact Tests: Detailed Report.

[48] Banerjee B. An Evaluation of Plastic Flow Stress Models for the Simulation of High-Temperature and High-Strain-Rate Deformation of Metals. arXiv.

[49] Steinberg D.J., Lund C.M. (1989). A Constitutive Model for Strain Rates from 10^−4^ to 10^6^ s^−1^. J. Appl. Phys..

[50] Nix W.D., Gibeling J.C., Hughes D.A. (1985). Time-Dependent Deformation of Metals. Metall. Trans. A.

[51] Tong W., Clifton R.J., Huang S. (1992). Pressure-Shear Impact Investigation of Strain Rate History Effects in Oxygen-Free High-Conductivity Copper. J. Mech. Phys. Solids.

[52] Tanner A.B., McDowell D.L. (1999). Deformation, Temperature and Strain Rate Sequence Experiments on OFHC Cu. Int. J. Plast..

[53] Jing C., Wang J., Zhang C., Sun Y., Shi Z. (2022). Influence of Size Effect on the Dynamic Mechanical Properties of OFHC Copper at Micro-/Meso-Scales. Int. J. Adv. Manuf. Technol..

